# New approaches to eliciting protective immunity through T cell repertoire manipulation: the concept of thymic vaccination

**DOI:** 10.1186/1476-9433-3-2

**Published:** 2004-12-08

**Authors:** Masha Fridkis-Hareli, Ellis L Reinherz

**Affiliations:** 1 Laboratory of Immunobiology, Department of Medical Oncology, Dana-Farber Cancer Institute, USA; 2 Department of Medicine, Harvard Medical School, 77 Avenue Louis Pasteur, Boston, MA 02115, USA

## Abstract

Conventional vaccines afford protection against infectious diseases by expanding existing pathogen-specific peripheral lymphocytes, both CD8 cytotoxic effector (CTL) and CD4 helper T cells. The latter induce B cell maturation and antibody production. As a consequence, lymphocytes within the memory pool are poised to rapidly proliferate at the time of a subsequent infection. The "thymic vaccination" concept offers a novel way to alter the primary T cell repertoire through exposure of thymocytes to altered peptide ligands (APL) with reduced T cell receptor (TCR) affinity relative to cognate antigens recognized by those same TCRs. Thymocyte maturation (i.e. positive selection) is enhanced by low affinity interaction between a TCR and an MHC-bound peptide in the thymus and subsequent emigration of mature cells into the peripheral T lymphocyte pool follows. In principal, such variants of antigens derived from infectious agents could be utilized for peptide-driven maturation of thymocytes bearing pathogen-specific TCRs. To test this idea, APLs of gp_33–41_, a D^b^-restricted peptide derived from the lymphocytic choriomeningitis virus (LCMV) glycoprotein, and of VSV8, a K^b^-restricted peptide from the vesicular stomatitis virus (VSV) nucleoprotein, have been designed and their influence on thymic maturation of specific TCR-bearing transgenic thymocytes examined *in vivo *using irradiation chimeras. Injection of APL resulted in positive selection of CD8 T cells expressing the relevant viral specificity and in the export of those virus-specific CTL to lymph nodes without inducing T cell proliferation. Thus, exogenous APL administration offers the potential of expanding repertoires *in vivo *in a manner useful to the organism. To efficiently peripheralize antigen-specific T cells, concomitant enhancement of mechanisms promoting thymocyte migration appears to be required. This commentary describes the rationale for thymic vaccination and addresses the potential prophylactic and therapeutic applications of this approach for treatment of infectious diseases and cancer. Thymic vaccination-induced peptide-specific T cells might generate effective immune protection against disease-causing agents, including those for which no effective natural protection exists.

## Introduction

Vaccination has improved healthcare by providing the most cost effective means to prevent disease on a global basis [1,2]. Since the first safe vaccine against smallpox infection was introduced by Sir Edward Jenner more than 200 years ago [3], a myriad of killed or live viral and bacterial vaccines as well as subunit (i.e. component) vaccines have been developed and proven to be highly effective [2]. The traditional approach to vaccine development from the early 1950's until today has been based most commonly on administration of weakened versions of disease-causing agents or certain of their components with appropriate adjuvants. In this way, successful vaccines against key viruses that cause acute infectious diseases of childhood (e.g. poliovirus, measles virus, mumps, rubella, chicken pox, etc.) have been developed. These vaccines induce peripheral T and B lymphocyte memory responses, affording protection against any future attack by disease-causing agents should it occur.

**Figure 1:**
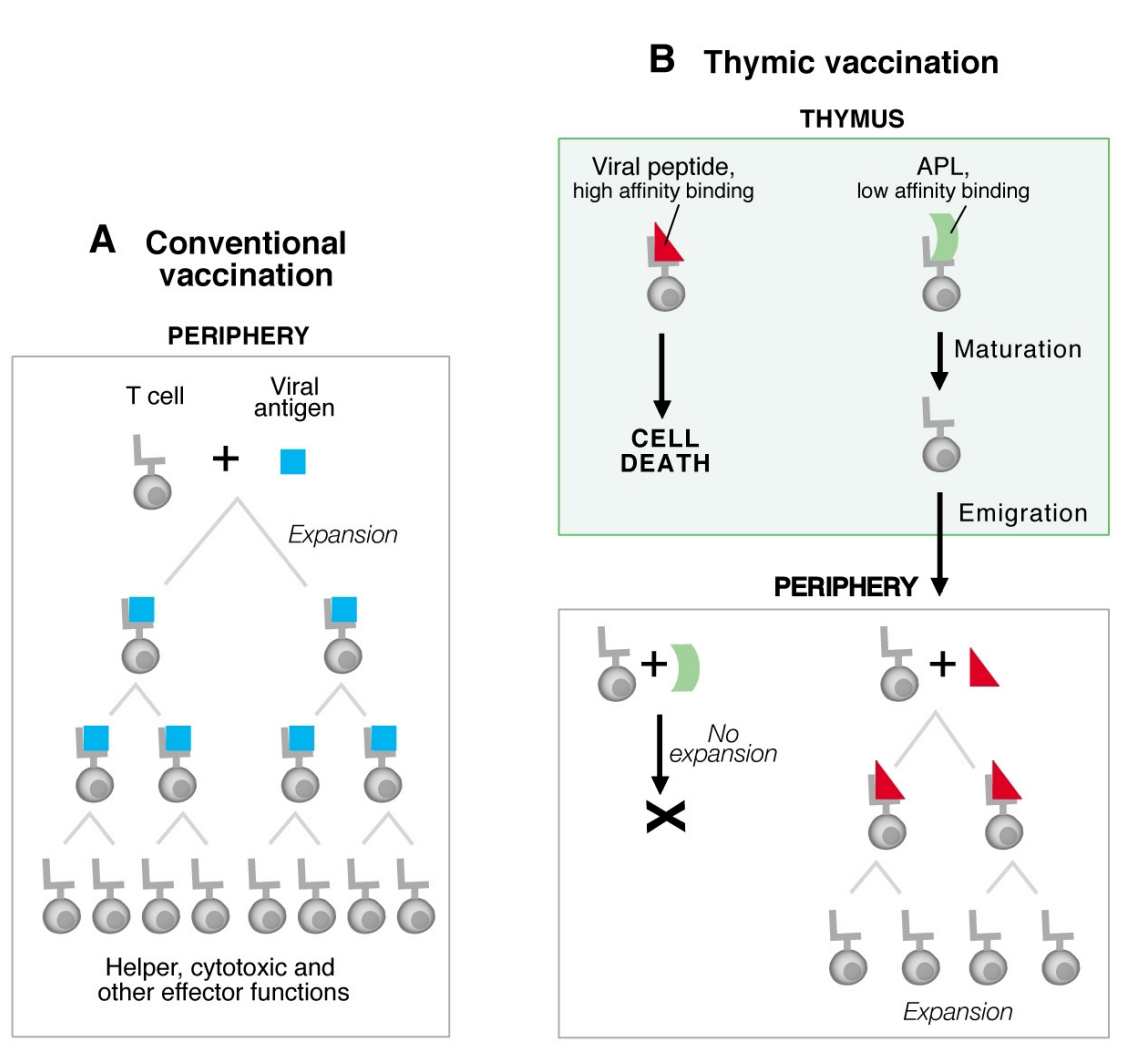
Thymic vaccination versus conventional vaccines. A. Conventional vaccines act on the mature peripheral lymphoid pool, in particular expanding existing T cells directed against the immunogen (blue square) derived from the disease-causing agent. Following subsequent infection, the T cell recognizes the pathogen, proliferates, mediates effector function and cytokines leading to immune response and elimination of the disease. For simplicity, the B lymphocyte response is not shown. B. Thymic vaccination offers a way to alter the primary T cell repertoire through exposure of immature thymocytes to APL with reduced TCR affinity relative to cognate antigens recognizing those TCRs. Thymocyte maturation (i.e. positive selection) is enhanced by the low affinity interaction between a TCR and an MHC-bound APL (green ribbon) in the thymus, with subsequent emigration of mature cells into the peripheral T lymphocyte pool. Those peripheral T cells can respond to cognate antigen (red triangle). Thus, variants of cognate antigens derived from infectious agents, tumors, etc. could be employed for peptide-driven maturation of thymocytes bearing pathogen-specific TCRs.

To date, the fundamental principles of vaccination have remained unchanged. The overriding concept for each vaccine has been the establishment of protective immunity largely due to antigen-specific T cell expansion, facilitating subsequent proliferation and differentiation of CD8 cytotoxic effector T cells and CD4 helper T cells capable of producing antiviral cytokines and chemokines (Fig. 1A). CD4 T cells activate B cells to generate neutralizing antibodies, offering protection against viral attachment/translocation or bacterial toxins, etc. [4,5]. As neutralizing antibodies have been the subject of recent reviews [6-9], they will not be considered further here.

However, conventional vaccines have their pitfalls. Microorganisms including HIV and malaria, among others, may alter their antigenic proteins through rapid mutagenesis, thereby hindering cytotoxic T lymphocyte (CTL)-based immunity, exploiting holes in the T cell repertoire, and/or misdirecting both cellular and humoral responses away from key cell-binding receptors to pathogen components which cannot provide epitopes for neutralizing antibodies [10-13].

The fundamental ways in which the immune system recognizes and responds to antigen are identical, irrespective of the source of molecules; microbes, allografts, allergens, autoantigens, or tumor antigens are approached in a similar manner. It follows that immune-based therapies that focus on promoting the quantity and quality of the immune response should be beneficial in the treatment of a range of diseases, especially persistent viral infections and cancer. Finding ways to increase the pool of mature, primed T cells that are able to fend off disease is a goal for future vaccine development. In this respect, a novel strategy for vaccine design, termed "thymic vaccination" has been considered to alter the T cell antigen receptor repertoire centrally via altered peptide ligands (APL). APL derived from infectious agents or tumor antigens, with low affinity to the TCR could, in principle, mediate positive selection and export of specific T cells from the thymus [14]. As such, these APL might be candidates for manipulating the thymic repertoire *in vivo*, controlling the generation of naive T cells and hence, subsequent memory development within the peripheral lymphoid compartment. Through repertoire manipulation, it should be possible to sculpt the specificity and diversity of disease-fighting cells. This thymic vaccination approach aims to deliver, by parenteral administration, positively selecting APL of cognate antigens into the thymus, eliciting maturation of thymocytes with desired TCR specificities at the level of thymic repertoire development (Fig. 1B). Note how engendering T cells with anti-viral specificity requires administration of an APL, a weaker affinity ligand for a given TCR to encourage maturation and emigration from the thymus.

Expanding T cell repertoires has enormous potential in aiding the organism's fight against infections or in affording tumor immunity. This strategy is principally different from conventional "peripheral" vaccination, which leads to proliferation of pre-existing mature T cells but does not alter the repertoire through creation of T lymphocytes with new T cell specificities. Thymic vaccination, by contrast, will alter the thymic repertoire to create desired T cell specificities. Moreover, a key feature of thymic vaccination is that it should be capable of directing the immune response towards those non-mutable components of proteins derived from infectious agents and tumors and away from misguiding cues that are part of pathogen or cancer chicanery. The rationale for this approach and the advantages over traditional vaccines are described below.

## Discussion

### Generation of T cell repertoire

**Figure 2:**
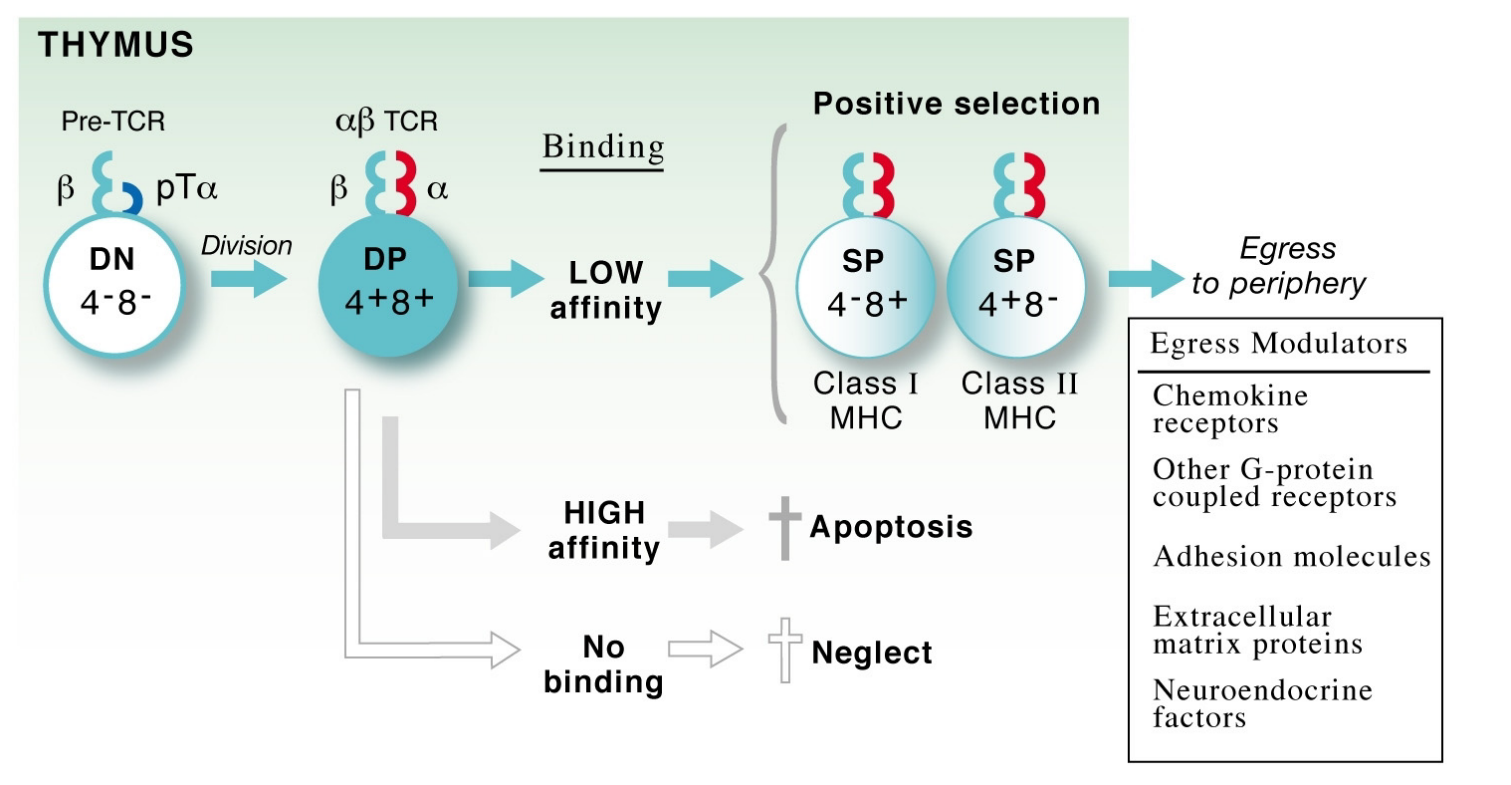
General scheme of thymocyte selection and emigration to the peripheral lymphoid compartment. CD4^-^CD8^- ^(DN) cells expressing pre-TCR undergo divisions and become αβTCR^+^CD4^+^CD8^+ ^(DP) at which stage they interact with self-peptides presented by class I and II MHC molecules expressed on thymic stromal cells. Those thymocytes whose TCRs interact with high affinity to pMHC undergo apoptosis, while those bound to pMHC with low affinity mature to become MHC class I-restricted CD8^+ ^SP or class II-restricted CD4^+ ^SP cells. These mature thymocytes then emigrate to the periphery aided by different egress-related mechanisms.

T cells bearing a highly diverse αβ T cell receptor (TCR) repertoire develop in the thymus from stem cells originating in the hemopoietic tissues [15-17]. On entering the thymus through the cortico-medullary junction, these cells migrate to the subcapsular epithelium and undergo a complex differentiation process in the thymic cortex and then in the medulla, involving proliferation, expression of accessory molecules, rearrangement of TCR genes and selection of the TCR repertoire (reviewed in [18,19]). T cell development does not occur autonomously but requires signals from non-hematopoietic stromal cells including various types of thymic epithelial cells (TECs) which show profound phenotypic differences between cortex and medulla. The thymic epithelium provides a broad spectrum of signals for thymocyte proliferation, differentiation and selection. Thymic nurse cells, expressing high levels of MHC class I and II molecules and also containing antigen processing machinery, are involved in thymocyte selection, mediated by peptide/MHC (pMHC) ligands. Pools of self-peptides bound to MHC molecules control both positive and negative selection (reviewed in [20]). Thymocytes that carry TCRs having low-affinity interactions with MHC-bound self-peptides are positively selected, and are exported into the pool of mature peripheral lymphocytes. In contrast, thymocytes bearing those TCRs that recognize self-peptides with "high" affinity are eliminated primarily upon interaction with dendritic cells [19,21]. A schematic representation of thymocyte development [DN (CD4^-^CD8^- ^double negative) → DP (CD4^+^CD8^+ ^double positive → SP (CD4^+^CD8^-^, CD4^-^CD8^+ ^single positive)] is depicted in Fig. 2.

### TCR/MHC/peptide interactions control thymocyte selection

The avidity of pMHC/TCR interactions plays a major role in T cell recognition [14]. Crystal structure analyses have revealed fine details about peptide conformation inside the peptide binding groove of MHC molecules and the amino acid residues interacting with the TCR Vα and Vβ domains including their CDR3 loops [22-25]. Peptide analogs of antigenic peptides with substitutes at amino acid residues, APL, have been shown to generate qualitatively different T cell responses compared with those produced by the antigenic peptides themselves [26]. Some APL act as TCR antagonists capable of positively selecting [27,28], negatively selecting [29], or otherwise altering [30] selection of thymocytes.

TCR-transgenic mice provide useful tools for studies of peptide-based thymocyte selection. For example, in N15 transgenic mice carrying a TCR specific for the vesicular stomatitis virus nucleoprotein octapeptide N_52–59 _(VSV8), VSV8 triggers negative selection of DP thymocytes in the context of H-2K^b^. In contrast, a weak agonist peptide variant, identical to the VSV8 peptide except for substitution of leucine for valine at the p4 peptide residue, termed L4, induces positive selection [31]. Similarly, in the P14 TCR transgenic mouse which expresses a TCR specific for the D^b^-restricted immunodominant LCMV epitope gp_33–41 _[32], the cognate gp_33–41 _peptide causes negative selection due to high affinity pMHC/TCR interactions. However, certain mutations of amino acid residues in the gp_33–41 _peptide affects the fate of thymocyte development in fetal thymic organ culture (FTOC) leading to positive selection P14-bearing thymocytes [33,34]. In yet a third TCR transgenic mouse model, F5, where the TCR recognizes a nucleoprotein peptide of the influenza virus NP_366–379 _in the context of H-2D^b^, a peptide antagonist mediates positive selection in FTOC [35,36], whereas the cognate peptide itself leads to deletion of DP thymocytes [37].

### Positive selection and emigration of antigen-specific thymocytes in vivo is mediated by APL of viral CTL epitopes

#### Design and initial characterization of APL derived from the viral epitopes gp_33–41 _and VSV8

**Figure 3:**
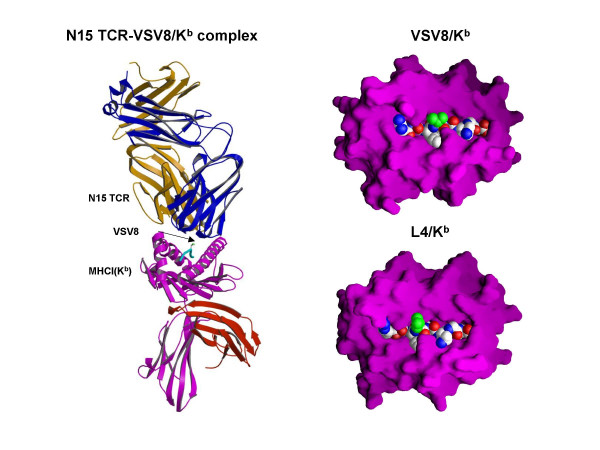
Structural basis of APL design. Crystal structure of N15 TCR-VSV8/K^b ^(left panel) [57]. The figure was rendered in MOLSCRIPT [58]. The TCR β chain is shown in gold, the TCR α chain in blue, K^b ^in magenta and β2M in red. Note the VSV8 peptide in green with the arrow pointing to the p4 valine side chain. Space-filling models of K^b ^in complex with VSV8 peptide and with its L4 variant (right panel) [31]. The K^b ^is shown as a GRASP surface [59] in magenta with peptide in CPK format and p4 residue atoms in green.

To experimentally test the concept of thymic vaccination, we have designed variants of gp_33–41 _and VSV8 peptides with substitutions at residues interacting with the TCR aimed at reducing TCR-pMHC affinity via diminution of the number of atomic contacts between the peptide and the TCR. Subsequently, we examined the effects of these APL on thymocyte maturation and emigration *in vivo *in two well-defined TCR-transgenic mouse systems. In the case of gp_33–41 _cognate peptide, amino acids at the peptide positions p4 (Tyr, Y) and p6 (Phe, F) were modified to Ser (S) and Ala (A), respectively, based on the crystal structure of the gp_33–41_/H-2D^b ^complex showing exposure of the side chains of these amino acid residues to the solvent and hence, TCR accessibility [38,39]. No change was made in the peptide anchor residues that occupy the binding pockets of H-2D^b^, thus ensuring proper peptide presentation in the context of MHC. In the other less extreme peptide variant of gp_33–41_, Ala (A) at p7 was substituted with Glu (E). For the VSV8 peptide, the weak L4 agonist with the substitution of Leu (L) for Val (V) at the p4 peptide residue has been employed. The crystal structure of the N15 TCR-VSV8/K^b ^complex as well as the space-filling models of K^b ^in complex with VSV8 and L4 peptides are shown in Fig. 3. The centrally positioned p4 peptide residue, whose atoms are shown in green in the space-filling model, faces up to the solvent and interacts with the N15 TCR. In spite of the subtle differences in the structure of VSV8/K^b ^as compared to L4/K^b^, these focal changes (p4 and Lys 66 on the α1 helix of H-2K^b^) determine the outcome of thymic selection [31]. In a similar way, D^b^/ gp_33–41 _vs. APL in which amino acid residues at p4 and p6 are altered, differentially affect development of thymocytes expressing the P14 TCR (data not shown). Experimental data using these APL (Y4S/F6A and A7E) for studies of thymocyte selection and emigration as applied to the thymic vaccination approach are summarized below (for the original work, see [40]).

The binding of the APL to the MHC class I molecules using RMA-S cells confirmed that amino acid substitutions at peptide residues interacting with the TCR did not affect peptide binding and, by extension, peptide presentation to T cells. A series of experiments was next performed to evaluate the functional potential of the APL to stimulate peripheral T cells in mice injected with the variant peptides. Results of both proliferation and cytokine secretion assays using mature T cells from P14Rag2^-/- ^lymph node and spleen showed a response to the high TCR affinity cognate peptide gp_33–41_, but not to the Y4S/F6A variant peptide. In support of this observation, tetramers of H-2D^b ^in complex with the Y4S/F6A peptide did not bind to SP CD8 thymocytes from P14 Rag2^-/- ^mice at any tetramer dilution, as judged by immunofluorescence analysis, whereas high fluorescence intensity staining was detected using tetramers of H-2D^b ^in complex with the gp_33–41 _peptide. The A7E/H-2D^b ^tetramer gave intermediate staining. These data suggested that the Y4S/F6A mutant must interact with the P14 TCR with extremely weak affinity, if at all.

#### Effect of APL administration on thymocyte development in vivo

Injection of the cognate viral peptides gp_33–41 _and VSV8 leads to negative selection of P14- and N15TCR-bearing thymocytes, respectively, due to relatively high affinity pMHC/TCR interactions [31,34]. *In vivo *administration of gp_33–41 _in P14Rag2^-/- ^mice and VSV8 injection into N15Rag2^-/- ^mice resulted in pronounced depletion of DP thymocytes. Surprisingly, injection of P14 Rag2^-/- ^mice with the Y4S/F6A peptide mutant resulted in a significant increase in the total number of thymocytes as well as the DP thymocyte subpopulation, while the A7E variant had no effect on thymocyte counts. As with Y4S/F6A in the P14Rag2^-/- ^system, injection of L4 in the N15Rag2^-/- ^mouse preserved the DP thymocytes and led to an increase in total thymocyte counts. The unusual increase in the number of DP thymocytes following exposure to Y4S/F6A peptide was not due to cellular proliferation and attendant DNA synthesis as examined by *in vivo *BrdU incorporation assay. Rather, Y4S/F6A peptide administration prevented apoptosis as confirmed by staining of thymocytes with anti-Annexin V mAb. To more directly test this hypothesis, we injected P14 Rag2^-/- ^mice with mixtures of the negatively selecting cognate peptide gp_33–41 _plus the Y4S/F6A APL. Increasing the amount of Y4S/F6A peptide in the injection mixture resulted in a higher number of total and DP thymocytes. Thus, we infer that the Y4S/F6A variant may compete with other endogenous negatively-selecting peptides for binding to H-2D^b ^molecules expressed on thymic stroma either by binding to "empty" surface MHC class I molecules or, perhaps, by a cross-presentation mechanism [41]. That A7E fails to afford positive selection and interacts significantly with the P14 TCR in D^b^/A7E tetramer binding assays suggest that this APL does not reduce TCR binding affinity sufficiently to stimulate positive selection.

#### Y4S/F6A and L4 peptides mediate positive selection and emigration of thymocytes in irradiation chimeras

The numerically small population of antigen-specific recent thymic emigrants (RTE) makes thymic selection/emigration studies difficult even with the use of TCR transgenic mice. To resolve this issue, we employed irradiation chimeras of congenic mouse strains (expressing the CD45.1 marker in B6 and CD45.2 in P14 and N15 transgenic mice) to determine whether interactions between the low affinity ligands, Y4S/F6A and L4, and their specific TCRs would result in thymic positive selection and subsequent emigration from the thymus. For this purpose, lineage-minus BM precursors of P14 – or N15- TCR transgenic Rag2^-/- ^mice (donor) were injected into irradiated congenic B6 mice (recipient) and the development of donor-type cells was monitored weekly by immunofluorescence staining and multicolor FACS analysis [40]. Following determination of the parameters related to the time period of appearance and the number of donor-type T cells in the chimeric thymus we administered the APL to the recipients at 3–4 wks after donor BM injection and assessed whether such exposure might influence the subsequent selection and emigration processes of donor thymocytes.

The numbers of donor DP and SP CD8^+ ^thymocytes in irradiation chimeras injected with Y4S/F6A were greatest, suggesting that this ligand mediated positive selection of P14 Rag2^-/-^-specific T cells. Similarly, in N15 Rag2^-/-^-B6 irradiation chimeras injected with L4, the numbers of both DP and SP CD8 donor thymocytes were highest, consistent with positive selection. In contrast, injection of either gp_33–41 _or VSV8 cognate viral peptides into irradiation chimeras led to thymocyte depletion by negative selection. Analysis of the peripheral lymphoid organs in these chimeras by triple color immunofluorescence with anti-CD45.2, anti-CD8α and anti-TCR-specific mAbs showed the greatest number of donor-type CD45.2^+^CD8^+^Vα2^+ ^T cells in the lymph nodes of Y4S/F6A -injected chimeras (2–3 fold over PBS-injected control mice), suggesting that donor-type thymocytes expressing the P14 TCR had developed in the presence of Y4S/F6A, matured and emigrated to the lymph nodes. A similar increase in the donor cell numbers were observed up to 9 weeks after injection of Y4S/F6A peptide. Positive selection was also evident in N15 Rag2^-/- ^-B6 irradiation chimeras injected with the L4 variant. In this case, higher CD8^+^Vβ5.2^+ ^N15 TCR transgene donor-type T cell numbers were observed both in lymph nodes and spleens. The functional analysis of donor-type CD8^+ ^lymph node T cells in irradiation chimeras injected with the positively selecting Y4S/F6A or L4 peptides showed approximately two-fold higher proliferation levels in response to the cognate peptides gp_33–41 _and VSV8, respectively, *in vitro*, compared to cells from PBS control-injected chimeric mice, reflecting the two-fold difference in the number of donor-type CD8^+ ^T cells in lymph nodes of chimeras injected with the APL. However, importantly, these mature donor-type T cells did not proliferate in response to Y4S/F6A or L4 variant peptides *in vivo*. Injection of the viral peptides and their APLs *in vivo *led to reduction of CFSE^+ ^staining in the case of gp_33–41 _and VSV8, suggestive of proliferation and/or activation-induced cell death (AICD). In contrast, no change in CFSE^+ ^staining was observed upon injection of Y4S/F6A or L4 peptides, implying that these APLs do not facilitate T cell expansion *per se*. In sum, the cognate peptide ligands gp_33–41 _and VSV8 which interact with the TCR with relatively high affinity compared to their respective APL, induce activation of peripheral T cells, whereas peptide variants Y4S/F6A and L4, which bind TCR with low affinity and mediate positive selection, do not stimulate mature T cell divisions.

### Significance of APL-driven T cell emigration for the thymic vaccination approach

The data described above and previously [40] represent the first examination of the direct effects of amino acid substitutions at the P14 and N15 TCR contact residues on thymocyte selection and emigration *in vivo*. In addition, we show that thymocyte emigration is dependent on the affinity/avidity of pMHC/TCR interactions. These results suggest that although the low affinity pMHC/TCR interactions are insufficient to trigger cell divisions in mature cells, differentiation of immature thymocytes nevertheless follows. Affinity measurements support the idea that positively selecting peptide ligand affinities are lower than those of negatively selecting ligands for TCRs, but additionally linked to their MHC binding/stability properties [42]. Our report is consistent with the notion that weak pMHCI/TCR interactions promote positive selection of SP CD8 thymocytes. Certainly the 10,000 fold weaker functional stimulation of N15-bearing T cells by L4 versus VSV8 peptide is in line with the view [14]. Two recent studies in class II MHC-restricted TCR transgenic mouse systems also argue that weak pMHC ligands may foster positive selection [43,44].

Collectively, our data show that cognate peptides can be modified at key TCR recognition positions to create variants that result in selection, directly or indirectly, of desired TCR specificities at the level of thymic development. This exogenous peptide administration offers a potential of expanding repertoire generation *in vivo *in a manner useful to the organism. Whether these peptide-specific T cells generate stronger defense mechanisms to fight viral infection or tumors in normal, non-transgenic mice remains to be investigated. The magnitude of the APL-driven increase in thymocytes and subsequent egress is only 2–3 fold, however. This level of change likely reflects the tightly regulated thymocyte egress process. In this respect, exploring peptide-based means of enhancing differentiation of thymocytes bearing desired TCRs together with the modulation of mechanisms controlling thymocyte emigration to the periphery would be of a great importance. To this end, various pathways regulating egress from the thymus are described below and should be considered as potential targets for such manipulation in conjunction with APL administration. Although not discussed further here, thymic vaccination followed by conventional cognate antigen immunization may be the best way to insure a robust memory T cell response.

### Regulation of thymocyte egress

Lymphocyte migration plays an important role in regulating the localization and orchestration of immune responses. As thymocytes progress through the developmental stages, they migrate from the cortico-medullary junction, the site of entry of T cell progenitors from the BM, to the subcapsular region of the thymus, then to the cortex and to the medulla [18,19]. Finally, functionally mature thymocytes exit the thymus and seed the peripheral lymphoid tissues. The processes that regulate trafficking of lymphoid precursors to and within the thymus, and that mediate emigration of mature T cells from the thymus to the periphery remain poorly understood. Several mediators, including chemokine receptors [45], adhesion molecules [46], extracellular matrix proteins [47], neuroendocrine factors [48] and G-protein coupled receptors (GPCR) [49] have been shown to regulate thymocyte export (Fig. 2). Recently, a role for the early activation marker CD69, transiently expressed on activated mature T cells and on thymocytes undergoing positive selection, in controlling thymocyte export, has also been suggested [50]. Cellular mechanisms involved in thymocyte egress are discussed in the following sections.

#### Chemokine pathways (reviewed in ref. [45,51,52])

Chemokines are basic polypeptides of about 100 amino acids, usually containing four Cys residues linked by disulphide bonds, which are produced by certain thymic stroma cells and are abundantly expressed in the thymus. Specifically, thymic epithelial, medullary epithelial and dendritic cells have been shown to secrete various chemokines. Growing evidence suggests that chemokines and their receptors, expressed differentially on thymocytes during discrete maturational stages, control homing of T cell progenitors to the thymus, their intrathymic migration, and exit to the periphery. Chemokines deliver signals for lymphocyte proliferation and survival, and regulate thymocyte trafficking by functioning in concert with other adhesion molecules such as selectins and integrins. Chemokines stimulate responding cells by activating pertussis toxin-sensitive G_i_α protein-coupled seven-transmembrane receptors (GPCR), leading to activation of intracellular secondary mediators which control directional cell migration. To date, 43 human chemokines have been identified, acting via binding to 19 different GPCR.

Some chemokine receptors are expressed in DP and SP thymocytes, e.g. CCR9, with its ligand CCL25 secreted by TEC and DC. Others, e.g. CCR5 and CCR8, expressed on mature SP thymocytes, have been suggested to play a role in mediating thymocyte emigration. In particular, CCR7 has been demonstrated to mediate homing of naïve T cells to peripheral lymphoid organs via ligands CCL19 and CCL21.

#### Extracellular matrix proteins (reviewed in [47])

Extracellular matrix (ECM) proteins laminin and fibronectin are produced by TECs, fibroblasts and MHC class II^+ ^macrophages in the thymus. Other ECM proteins including nidogen, associated with laminin, and galectins -1, -3, and -5 as well as glycosaminoglycans are produced by thymic epithelium. ECM proteins form molecular bridges between thymocytes and the thymic microenvironment, mediating adhesion of thymocytes via their ECM receptors VLA-4, -5 and -6, and their disassembly from the cell complexes. In the absence of ECM proteins, normal thymocyte development and migration are severely perturbed, both in *in vitro *cultures of TEC and in *in vivo *knockout mouse models, suggesting a crucial role of the ECM protein network in the thymic function.

#### S1P pathway (reviewed in [53,54])

Sphingosine 1-phosphate (S1P), a member of sphingolipid family, is an important signaling molecule present in high concentrations in body fluids. SIP binds to members of a family of G protein-coupled receptors (S1P_1–5_/Edg) with up to nanomolar affinity, triggering diverse effects, including proliferation, survival, migration, morphogenesis, adhesion molecule expression, and cytoskeletal changes. S1P receptors are widely expressed during embryonic development and in the adult. The tissue distribution shows that lymphoid organs express high levels of S1P_1 _and S1P_4_. Thus, these receptors may be potential targets for pharmacological drug design aimed at effecting thymocyte migration.

The expression of S1P_1 _on T cells controls their exit from the thymus and entry into the blood, and, thus, has a central role in regulating the numbers of peripheral T-cells [55]. Interestingly, S1P_1 _knock-out mice show a block in the egress of mature T-cells into the periphery. The regulated expression of S1P_1 _receptor levels, which is increased in mature SP thymocytes and peripheral T cells, may control responsiveness to the high levels of sphingosine 1-phosphate in the blood, which selectively induces mature T-cell migration to the periphery. Recently, S1P_1 _receptors have been implicated in lymphocyte trafficking and homing based on studies using FTY720, a potent immunosuppressive agent, which is an agonist ligand for S1P_1,3,4,5 _receptors blocking egress of T cells from the thymus. Studies of thymocyte egress mechanisms through the S1P receptor pathway may aid in facilitating emigration from the thymus to the periphery and provide additional means of enriching the mature T cell pool with desired specificities.

### Therapeutic applications of thymic vaccination

Currently available vaccines unquestionably represent a success story in modern medicine and have had a dramatic effect on morbidity and mortality worldwide. Nonetheless, it is clear that improvements are required to enable the development of vaccines against infectious diseases that have so far proven difficult to control with conventional approaches (HIV-1, malaria, tuberculosis, etc.).

Thymic vaccination might offer promising clinical applications as a way of immunization against these infectious diseases and cancers, enabling "designer" thymic development to produce suitable and long-lasting protective T cell immune specificities. In a converse role, deletion of unwanted T cell specificities in the case of autoimmunity by agonist administration early in life could be considered. Assuming thymic vaccination proves clinically viable, immune responses against invariant components of infectious agents, such as HIV and malaria, which otherwise utilize their intrinsic mutational capacity to evade human immune recognition, can be targeted. Design of novel vaccines based on the thymic vaccination approaches will benefit from the information gained in the recently completed Human Genome Project, particularly as genetic polymorphism associated with high risk of developing certain diseases later in life including cancers, infectious disease susceptibility and autoimmunity are uncovered. For example, the ability to manipulate the T cell repertoire to elicit anti-tumor responses early in life may prevent clinical disease evolution later. Powerful bioinformatic tools such as computer-based identification of HLA-allele specific binding epitopes and structural insight into TCR-pMHC interactions will aid in the epitope-based APL design process [56].

## Conclusions

A thymic vaccination strategy has been conceived based on the current knowledge of thymocyte differentiation and repertoire generation. This approach differs substantially from conventional vaccination since it aims to shape T cell responses through thymic repertoire manipulation, exposing developing thymocytes to positively selecting APL derived from infectious agents or tumors. Experimental data to date suggest that this strategy is possible in *in vivo *mouse models using irradiation chimeras reconstituted with bone marrow progenitors from TCR-transgenic animals. Increasing emigration of antigen-specific T cells from the thymus to the periphery is a challenging goal. In the future, a combined approach of exposing the subject to a positively selecting APL plus a thymic export-enhancing agent might generate practical and efficient protective repertoire manipulations. Potential applications may include design and administration of APL against cancer, infectious and autoimmune diseases.

## List of abbreviations

APL, altered peptide ligand; BM, bone marrow; CMJ, cortico-medullary junction; DC, dendritic cells; DN, double negative; DP, double positive; GPCR, G-protein-coupled receptors; HEV, high endothelial venule; MMP, matrix metalloproteinases; RTE, recent thymic emigrants; SP, single positive; S1P, sphingosine 1-phosphate; TCR, T cell receptor; TEC, thymic epithelial cells.

## Competing interests

The author(s) declare that they have no competing interests.

## Authors' contributions

MFH carried out the study, including experimental design and data acquisition, drafted and revised the manuscript. ELR conceived of the study, participated in its design and coordination, and helped to draft and revise the manuscript. The authors read and approved the final manuscript.
